# Drought Responses in Poaceae: Exploring the Core Components of the ABA Signaling Pathway in *Setaria italica* and *Setaria viridis*

**DOI:** 10.3390/plants13111451

**Published:** 2024-05-23

**Authors:** Isabella Peres de Oliveira, Camila Schaaf, Nathalia de Setta

**Affiliations:** Centro de Ciências Naturais e Humanas, Universidade Federal do ABC, São Bernardo do Campo 09606-045, SP, Brazil; oliveira.isabella@aluno.ufabc.edu.br (I.P.d.O.); camila.schaaf@ufabc.edu.br (C.S.)

**Keywords:** *SnRK2*, *PP2C*, *PYL/PYR/RCAR*, genome-wide, meta-analysis, gene expression

## Abstract

Drought severely impacts plant development and reproduction, reducing biomass and seed number, and altering flowering patterns. Drought-tolerant *Setaria italica* and *Setaria viridis* species have emerged as prominent model species for investigating water deficit responses in the Poaceae family, the most important source of food and biofuel biomass worldwide. In higher plants, abscisic acid (ABA) regulates environmental stress responses, and its signaling entails interactions between PYR/PYL/RCAR receptors and clade A PP2C phosphatases, which in turn modulate SnRK2 kinases via reversible phosphorylation to activate ABA-responsive genes. To compare the diversity of *PYR/PYL/RCAR*, *PP2C*, and *SnRK2* between *S. italica* and *S. viridis*, and their involvement in water deficit responses, we examined gene and regulatory region structures, investigated orthology relationships, and analyzed their gene expression patterns under water stress via a meta-analysis approach. Results showed that coding and regulatory sequences of *PYR/PYL/RCARs*, *PP2Cs*, and *SnRK2s* are highly conserved between *Setaria* spp., allowing us to propose pairs of orthologous genes for all the loci identified. Phylogenetic relationships indicate which clades of *Setaria* spp. sequences are homologous to the functionally well-characterized *Arabidopsis thaliana PYR/PYL/RCAR*, *PP2C*, and *SnRK2* genes. Gene expression analysis showed a general downregulation of *PYL* genes, contrasting with upregulation of *PP2C* genes, and variable expression modulation of *SnRK2* genes under drought stress. This complex network implies that ABA core signaling is a diverse and multifaceted process. Through our analysis, we identified promising candidate genes for further functional characterization, with great potential as targets for drought resistance studies, ultimately leading to advances in Poaceae biology and crop-breeding strategies.

## 1. Introduction

Abscisic acid (ABA) phytohormone plays a crucial role in plant signaling. It has an ancient origin in evolutionary history [1], being present in all vascular plants, as well as some species of cyanobacteria, algae, bryophytes, fungi, and lichens [2]. ABA orchestrates various biochemical and physiological processes [3], such as vegetative growth, seed dormancy and germination, flowering and fruit ripening, leaf senescence, osmotic regulation, and stomatal closure [4,5,6]. ABA also governs responses to a wide range of environmental stresses, whether biotic or abiotic [7], through transcriptional and post-transcriptional mechanisms [8], modulating physiological changes at the cellular level, resulting in whole-system response and adaptation [9].

ABA signaling in higher plants involves the interplay between PYRABACTIN RESISTANCE 1/PYR1-LIKE/REGULATORY COMPONENTS OF ABA RECEPTORS (PYR/PYL/RCARs) receptors and class A of PROTEIN PHOSPHATASE TYPE 2Cs (PP2Cs), which, in turn, modulate SNF-related protein kinase 2s (SnRK2s). In the absence of ABA, PP2Cs are active and inhibit SnRK2s by the dephosphorylation of activation sites [6]. Conversely, in the presence of ABA, the structure of PYR/PYL/RCAR receptors changes, allowing these proteins to bind to ABA and PP2Cs, inhibiting PP2Cs and facilitating the accumulation of phosphorylated SnRK2s [6]. SnRK2 kinases can then activate its multiple downstream regulatory proteins, the ABA-responsive element binding factors (ABFs), which are essential for the activation of ABA-induced genes [4,6]. ABFs modulate ABA-dependent gene expression by binding to the cis-acting ABA-responsive elements (ABRE) located in the regulatory regions of the ABA-induced genes [10]. ABREs are extensively studied cis-elements involved in ABA-induced gene expression, functioning as transcription factor binding sites for ABA-responsive element binding factors (ABFs) [11]. Several ABREs, or combinations of ABREs with other cis-acting elements, are necessary for ABA-responsive transcription to take place [12,13].

The 14 *Arabidopsis thaliana* PYL receptors have different ABA-binding properties and selectively interact with PP2C co-receptors [14,15,16]. In general, PYL receptors are differentially expressed in multiple tissues and at different stages of plant development [17]. PP2C is a broad family of plant phosphatases divided into 12 subfamilies. Class A of PP2C phosphatases is especially responsive to ABA and ABA-related stress, consisting of nine protein-coding genes in *A. thaliana* [18]. The origin of the *SnRK2* gene family predates the split between green algae and the ancestor of land plants, emphasizing its significance in the adaptation of plants to land environments [19]. Through duplication and subsequent exon-intron loss events, *SnRK2* has diversified, acquiring distinct regulatory functions and responsiveness to ABA. Phylogenetic studies have categorized the 10 *A. thaliana SnRK2* members into subclasses I, II, and III, which have different roles in the response to the abiotic stress response [20].

Drought is one of the most important abiotic stresses impacting plant development and reproduction, reducing biomass production and seed number, as well as altering the morphology and time of flowering patterns [21,22]. Severe and irregular drought events are growing worldwide as global temperatures persistently increase. Extreme temperatures and shifting precipitation patterns are a major concern for crop production and global food security [23]. *Setaria italica* and *Setaria viridis* (*Setaria* spp.) have emerged as plant models to study drought response in the plants of the Poaceae family due to their small diploid genome, short life cycle, and fully sequenced genome availability. The Poaceae family, which includes valuable world crops like rice, wheat, maize, sorghum, and sugarcane, is a key plant family to both human and animal food production, as well as a biomass source for biofuel production. *Setaria viridis*, known as green foxtail, first emerged near the 45th parallel north in Eurasia before spreading to various temperate, tropical, and subtropical habitats across the globe. Its remarkable history of colonization and adaptation to local conditions, coupled with its simple growth necessities, has led to its reputation as one of the most problematic weeds in agriculture [24]. *Setaria italica*, also known as foxtail millet, was domesticated from its wild sibling species, *S. viridis*, more than 8000 years ago in northern China [25]. It is commonly grown in arid and semi-arid regions of China and India, having natural tolerance to low-water environments and great water usage efficiency [26]. Exploring the mechanisms through which *Setaria* spp. regulate gene expression, particularly the ABA pathway, in response to drought is of significant value to understand its resistance mechanisms. Such insights can be applied in enhancing abiotic stress tolerance not only in *Setaria* spp. but also in other major crops belonging to the same family.

In *Setaria* spp., genome-wide analyses of *PYR/PYL/RCAR*, *PP2C*, and *SnRK2* genes were exclusively conducted in *S. viridis* [4]. This study unveiled eight *PYL* receptor loci, divided into three subfamilies, as well as 12 *PP2Cs* and 11 *SnRK2s* loci. The *SnRK2s* loci were grouped according to the three *A. thaliana* subclasses. Expression patterns of *PYL* genes in *S. viridis* demonstrated either reduction or stability in shoots under ABA treatment, drought, and salt stress, contrasting with monocots such as *O. sativa* and *T. aestivum* [27]. In these monocots, at least half of the *PYLs* were found to be upregulated under ABA treatment [28,29]. Conversely, *PP2C* genes expression predominantly increased in shoots under drought, ABA treatment, and salt stress [4]. Regarding *SnRK2* loci, gene expression profiles showed less uniformity under osmotic stress in *S. viridis* [4], with some being mostly upregulated (*SvSnRK2.9*, *SvSnRK2.10*, and *SvSnRK2.11*) and others downregulated (*SvSnRK2.6*, *SvSnRK2.7*, and *SvSnRK2.8*).

In this study, we characterized the genes of *S. italica* from the *PYL/PYR/RCAR*, *PP2C*, and *SnRK2* gene families and identified their orthologues in *S. viridis*. The results showed a high conservation among sequences of these sibling species, with the most significant variation observed in their regulatory regions. Phylogenetic analyses enabled the clustering of *Setaria* spp. genes, *Oryza sativa*, and *A. thaliana*, species in which the function of PYL/PYR/RCAR, PP2C, and SnRK2 proteins have been more extensively studied. Lastly, we conducted a systematic meta-analysis of gene expression profiles in *Setaria* spp. under water stress conditions, enabling us to pinpoint genes modulated during water deficit responses.

## 2. Results

### 2.1. The Core ABA Signaling Genes of S. italica and S. viridis Are Highly Conserved

We identified 8, 12, and 11 encoding sequences for *S. italica PYR/PYL/RCARs*, *PP2Cs*, and *SnRK2s*, respectively (Table 1). The total *S. viridis* gene number validated earlier findings [4], with eight genes from the *PYR/PYL/RCAR* family, 13 genes from the *PP2C* family, and 11 genes from the *SnRK2* family. The number of loci for the three gene families exhibited high conservation between the two species, apart from the duplication of the *SvPP2C2* gene in *S. viridis*. *SnRK2s* showed longer transcripts (4418 ± 1018 bp), followed by *PP2Cs* (2986 ± 1149 bp) and *PYR/PYL/RCARs* (1686 ± 1065 bp). On the other hand, the longer amino acid sequences mean belonged to PP2C proteins (399 ± 64 aa), followed by SnRK2s (362 ± 31 aa) and PYR/PYL/RCARs (206 ± 17 aa).

**Figure 1:**
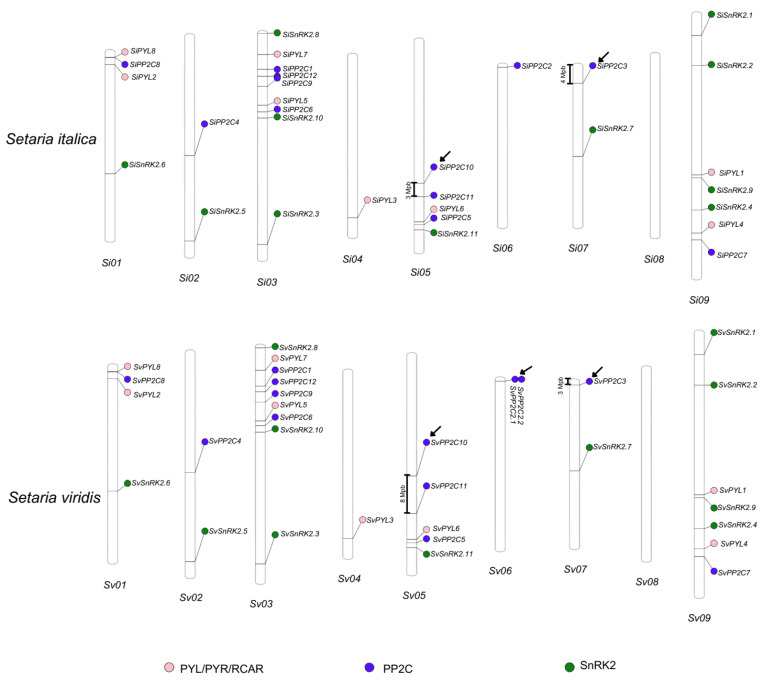
Chromosomal location analysis of the *S. italica* (**above**) and *S. viridis* (**below**) *PYR/PYL/RCAR*, *PP2C*, and *SnRK2* genes. The physical mapping of the genes was based on data retrieved from Phytozome. Chromosomes were drawn to scale and chromosome numbers were indicated below each chromosome ideogram. Pink, blue, and green circles represent *PYR/PYL/RCAR*, *PP2C*, and *SnRK2* gene families, respectively. Arrows indicate major differences between *S. italica* and *S. viridis* gene locations.

A comparative analysis of the chromosomal locations was performed to assess orthology relationships between genes of *S. viridis* and *S. italica*. The genes from all three families were spread across the nine chromosomes in both species, with a significant conservation of the chromosomal locations between the *Setaria* spp. orthologous gene pairs (Figure 1). Major differences among chromosomal locations were identified for the *PP2C* phosphatases. Firstly, *SvPP2C2* underwent tandem duplication (*SvPP2C2.1*: Sevir.6G004900 and *SvPP2C2.2*: Sevir.6G005000), with the genes positioned in inverted orientations. Secondly, the *PP2C10* and *PP2C11* genes were separated by about 3 Mb in *S. italica*, whereas the region separating these genes in *S. viridis* was considerably longer, spanning about 8 Mb in length. Lastly, on chromosome 7, the *PP2C3* gene of *S. italica* was positioned approximately 4 Mbp from the beginning of the chromosome, while in *S. viridis*, it was located about 3 Mbp from the beginning of the chromosome. 

**Figure 2:**
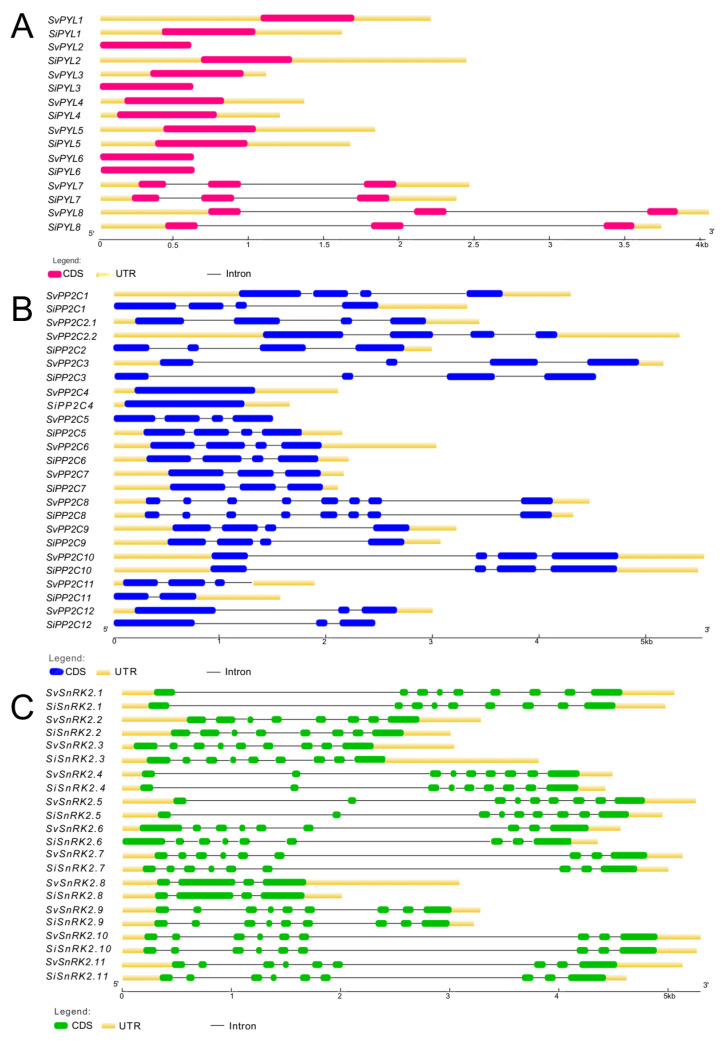
Exon–intron structures of the *S. italica* and *S. viridis* genes *PYR/PYL/RCAR* (**A**), *PP2C* (**B**), and *SnRK2* (**C**) coding sequences. Pink, blue, and green boxes represent *PYR/PYL/RCAR*, *PP2C*, and *SnRK2* coding sequences, respectively. Yellow boxes represent 5′ and 3′ UTRs, and black lines indicate introns. Gene structures were drawn to scale and oriented from 5′ to 3′ UTRs.

In order to compare the structures of *S. viridis* and *S. italica* genes, we drew exon-intron structures using the Gene Structure Display Server (GSDS) tool. Most of *S. viridis* genes were longer than *S. italica* orthogues (Figure 2), with the exception of *PYL2*, *PP2C5*, and *SnRK2.3*. The differences in the length of the transcribed regions were mainly due to 5′ and 3′ UTR lengths. In *S. italica*, several *PP2C* genes lacked 5′ UTR regions, including *SiPP2C1*, *SiPP2C2*, *SiPP2C3*, *SiPP2C11*, and *SiPP2C12*. The *SvPP2C2.1* duplicated gene had a closer gene structure to *SiPP2C2* than its paralogue, *SvPP2C2.2*. We also evaluated the coding and protein sequence identities among the orthologous *PYR/PYL/RCAR*, *PP2C*, and *SnRK2* sequences. As expected for sibling species, we observed a high degree of identity among orthologous pairs, especially for amino acid sequences (Table 2). The mean amino acid sequence identities ranged from 99.9 ± 0.2% for SnRK2 to 97.2 ± 9.0% for PP2C, while for nucleotide coding sequences, the mean values varied from 99.8 ± 0.2% for *SnRK2* to 97.2 ± 8.8% for *PP2C*. Most of the substitutions within the coding sequences were synonymous, and the indels did not disrupt the open reading frames (ORFs), thus not leading to significant differences in the protein sequences. The most divergent orthologous sequence pair was *SiPP2C2/SvPP2C2.2*, which included one of the duplicated *PP2C2* genes of *S. viridis*. This orthologous pair exhibited only 67.3% of amino acid and 67.9% of nucleotide identity. Most of these differences correspond to substitutions and indels in the first exon.

**Figure 3:**
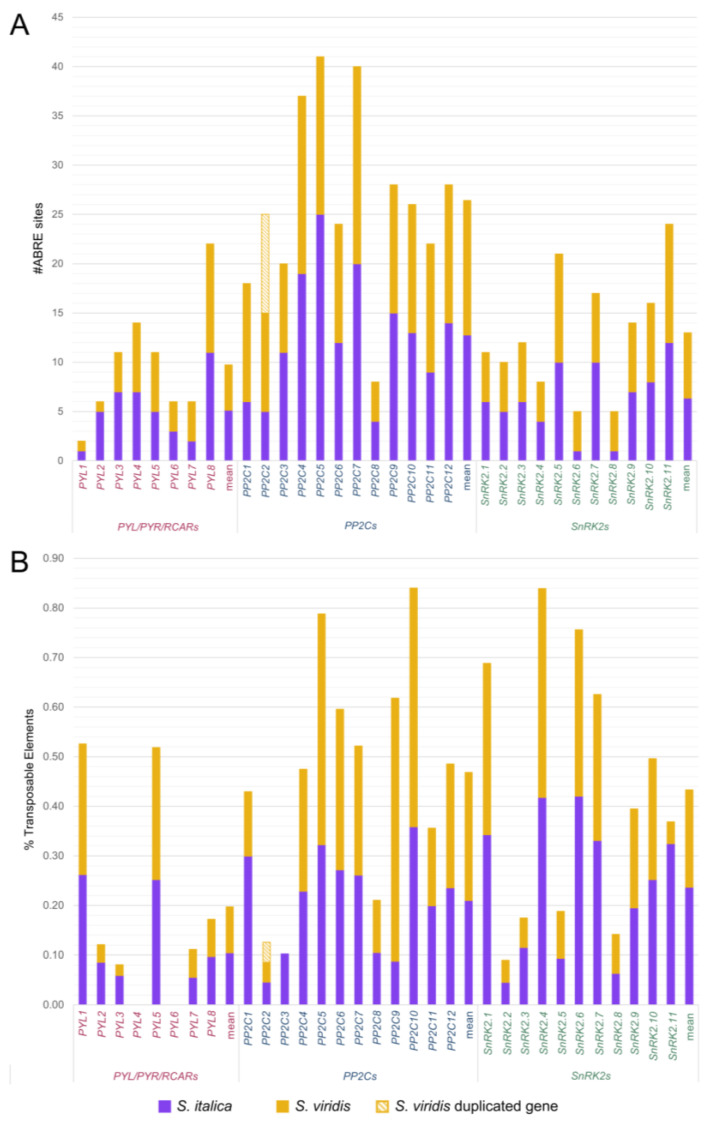
Analysis of the number of ABRE sites (**A**) and the proportion of transposable elements coverage (**B**) in the putative regulatory regions of *Setaria* spp. *PYR/PYL/RCAR*, *PP2C*, and *SnRK2* genes. *Setaria italica* and *S. viridis* orthologous gene pairs are grouped in the same bars. Purple and orange bars represent *S. italica* and *S. viridis* values, respectively.

In an attempt to identify whether *S. italica* and *S. viridis PYR/PYL/RCAR*, *PP2C*, and *SnRK2* genes can be differentially modulated in response to the ABA signaling pathway, the presence of cis-acting ABRE sites was surveyed in the putative regulatory regions. For this analysis, we defined as putative regulatory sequences DNA fragments of both species that encompass the longest 5′ UTR of each pair of orthologous genes, plus 2 kb upstream of the transcription start sites (TSSs) of each gene. Thus, the length of putative regulatory sequences analyzed varied from 2000 to 2665 bp for *PYL* receptors, from 2055 to 3160 bp for *PP2C* phosphatases, and from 2100 to 2595 bp for *SnRK2* kinases (Table S1). *PP2C* genes exhibited the highest mean number of ABRE sites, followed by *SnRK2s* and *PYLs*, with significant variation among genes within each family (Figure 3A). For example, while *PYL1s* have only one ABRE site, *PYL8s* showed 11 ABREs in its regulatory regions. On the other hand, the number of ABRE sites was very similar for *S. italica* and *S. viridis* species, with few exceptions such as the *SvSnRK2.6* and *SvSnRK2.8* genes, which had four times as many ABRE sites as *SiSnRK2.6* and *SiSnRK2.8*, respectively.

Transposable elements (TEs) are ubiquitous DNA sequences with the ability to transpose and replicate within the genomes of host species, thereby generating genetic variability linked to their activity. One way in which TEs generate genetic variability is by modulating gene expression, whether inserted within or near the genes, not only affecting the qualitative and quantitative production of proteins but also potentially impacting associated metabolic pathways [21,30]. To evaluate the genetic variability in the regulatory regions of *Setaria* spp. *PYR/PYL/RCAR*, *PP2C*, and *SnRK2* genes associated with TE insertions, we conducted an analysis to determine the TE coverage. We observed a higher percentage of TEs in *PP2C* phosphatases and *SnRK2* kinases than in *PYL* receptors, with two *SnRK2* sequences (*SnRK2.4* and *SnRK2.6*) showing more than 40% of TEs covering their putative regulatory sequences (Figure 3B). Despite some interesting patterns, there was no difference in the mean TE composition between the *S. italica* and *S. viridis* regulatory regions. However, among *PP2C* phosphatases, a higher frequency of genes containing more TEs was observed in *S. viridis* compared to *S. italica* (*PP2C5*, *6*, *9*, and *10*). Finally, three *PYL* and three *SnRK2* (*PYL2*, *PYL3*, *PYL8*, *SnRK2.3*, *SnRK2.6*, and *SnRK2.11*) sequences showed more TEs in *S. italica* than in *S. viridis*.

### 2.2. Phylogenetic Analyses Offer a Valuable Tool for Associating Setaria spp. Genes to Other Homologous Genes with Well-Defined Functions

**Figure 4:**
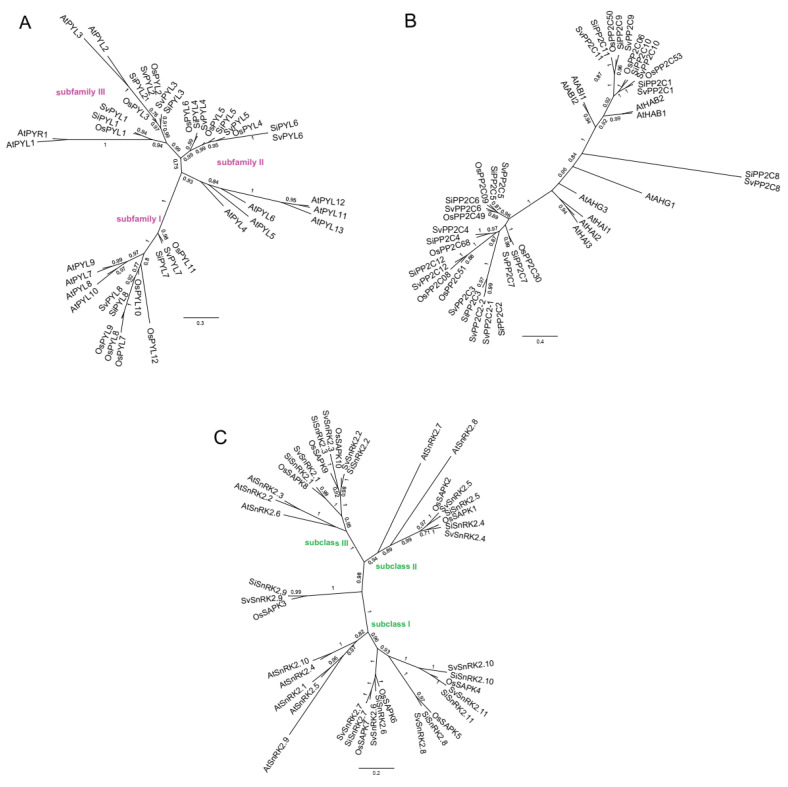
Phylogenetic analysis of *PYR/PYL/RCAR* (**A**), *PP2C* (**B**), and *SnRK2* (**C**) gene families. The unrooted trees were reconstructed using the PhyML tool and the maximum likelihood method. At: *A. thaliana*; Os: *O. sativa*; Si: *S. italica*; Sv: *S. viridis*. The numbers at each branch are the percentage occurrence values of the clusters calculated with the aLTR SH-like method. The *PYR*/*PYL*/*RCARs*, *PP2Cs*, and *SnRK2s* alignments were 498, 1203, and 1155 characters long, respectively. We indicated *PYL* subfamilies and *SnRK2* subclasses in pink and green, respectively.

To analyze the evolutionary history of the *PYR/PYL/RCAR*, *PP2C*, and *SnRK2* gene families in *Setaria* spp., unrooted phylogenetic trees were constructed based on the alignment of coding nucleotide sequences. The sequences of *A. thaliana* and *O. sativa*, previously analyzed by Duarte and colleagues [4], were also included in the trees. All trees reinforced the orthology relationships between the *Setaria* spp. genes (Figure 4). *Oryza sativa* and *A. thaliana* genes grouped basally to *Setaria* spp. sequences, in accordance with the phylogenetic relationships of the species. *PYL* receptor phylogeny grouped *Setaria* spp. sequences in three distinct clades (Figure 4A), or subfamilies, as previously proposed for *S. viridis* sequences [4]. Subfamilies I, II, and III harbored *Setaria* spp. *PYL7-8*, *PYL4-6*, and *PYL1-3*, respectively. Subfamilies II and III exhibited a close phylogenetic relationship. *Setaria* spp. subfamilies clustered with *A. thaliana PYLs* as follows: subfamily I with *AtPYL8-10*, subfamily II with *AtPYL4-6* and *AtPYL11-13*, and subfamily III with *AtPYR1* and *AtPYL1-3*. Sequences of *Setaria* spp. *PP2C* clustered into two distinct groups, with *PP2C8* positioned separately outside these clusters (Figure 4B). One cluster was basally connected by *AtABI1, AtABI2*, *AtHAB1*, and *AtHAB2*, while the other cluster was closer to *AtHAI1-3*. Finally, *SnRK2* phylogeny evidenced the three subclasses of *SnRK2* kinases (Figure 4C). Subclass I included *SnRK2.6*, *SnRK2.7*, *SnRK2.8*, *SnRK2.10*, and *SnRK2.11*; subclass II, *SnRK2.4* and *SnRK2.5*; while subclass III grouped *SnRK2.1*, *SnRK2.2*, and *SnRK2.3*. Different from the tree presented by Duarte and colleagues [4], our phylogeny placed *Setaria* spp. *SnRK2.9* basally.

### 2.3. Meta-Analysis of Gene Expression Supports a Diversity of Roles of ABA Core Components during the Response to Water Stress

Aiming to investigate the expression patterns of core ABA signaling genes under drought stress, a meta-analysis was conducted using data from studies involving water deficit and PEG-induced drought stress in *Setaria* spp. Literature searches were performed from October 2020 to February 2024 using Google Scholar [31] and PubMed [32] websites (Table S2). We were able to track eight papers that had LogFC data available for download and analysis (Figure S1) [21,33,34,35,36,37,38,39]. LogFC was used to quantify the level of modulation in gene expression between experimental and control conditions (hereafter referred to as the sample). No studies with available LogFC data were identified for *S. viridis*, leading to its exclusion from the subsequent analysis. A total of 24 samples were identified for *S. italica* (Table S3). 

**Figure 5:**
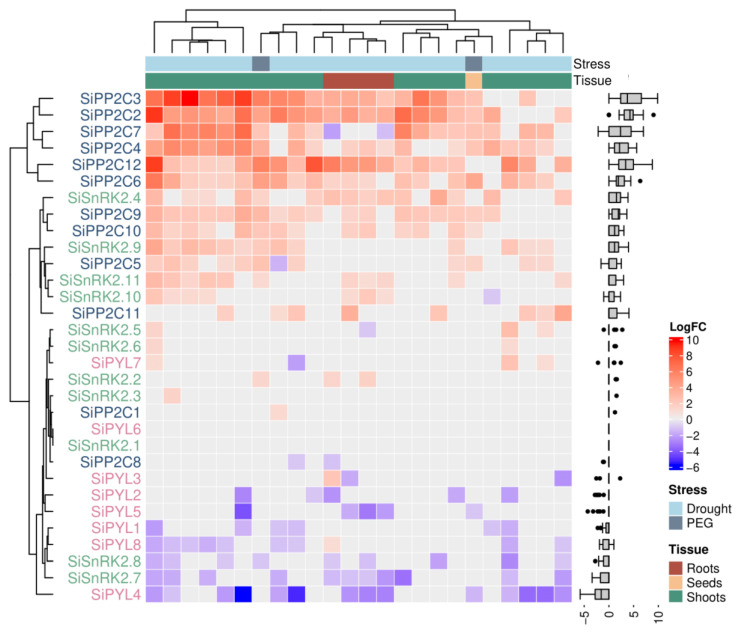
Heatmap of LogFC values for core ABA signaling genes. *PYR/PYL/RCAR*, *PP2C*, and *SnRK2* gene names are written in pink, blue, and green, respectively. Boxplots indicate the LogFC profiles for each gene. Dendrograms on the left and above the heatmap show the clustering patterns of genes and samples, respectively.

We observed a general downregulation of *PYL* genes, with the exception of *PYL7* (subfamily I), which displayed upregulation in three samples and downregulation in one, and *PYL6* (subfamily II), which exhibited no significant differential expression (Figure 5). Notably, *PYL4* (subfamily II) showed the most pronounced downregulation. Our analysis of seed tissue did not reveal upregulation in any *PYL*. Contrasting with the *PYL* expression profile, our meta-analysis unveiled a pattern of strong upregulation across *PPC2* genes, with the exception of *PP2C8* and *PP2C1*, which were downregulated in two samples and upregulated in only one sample, respectively (Figure 5). *PP2C3* emerged as the most upregulated gene, being clustered with the other strongly upregulated *PP2C2*, *PP2C4*, *PP2C6*, *PP2C7*, and *PP2C12*. However, it is worth noting that the expression of *PP2C7* in root tissue exhibited a contrasting downregulation. Lastly, the expression profiles of *SnRK2* genes displayed the greatest variability (Figure 5). *SnRK2.4*, *SnRK2.9*, *SnRK2.10*, and *SnRK2.11* showed an important upregulation. In contrast, *SnRK2.8* and *SnRK2.7* displayed expressive downregulation; *SnRK2.1* was not differentially expressed and, *SnRK2.2*, *SnRK2.3*, *SnRK2.5*, and *SnRK2.6* were weakly upregulated (Figure 5). 

## 3. Discussion

The genome and transcriptome sequencing of plant species has offered invaluable insights into genome functionality and the adaptive mechanisms these species employ under diverse environmental conditions. Sibling species may manifest distinct phenotypes due to subtle variations in protein products, differences in gene expression profiles, and post-transcriptional and post-translational modifications. Previous studies have demonstrated that regardless of the phenotypic variation and the wide global distribution of *S. viridis*, its populations display low genetic diversity [33,34]. The genetic diversity in *S. italica* is even more restricted, a pattern commonly seen in domesticated species [35]. Therefore, the low genetic variability observed for the *PYL/PYR/RCAR*, *PP2C*, and *SnRK2* gene families in *S. italica* and *S. viridis* was not unexpected. The main differences were associated with the presence and length of the UTRs, the number of ABRE elements, and the presence of TEs in the regulatory regions (Figure 2 and Figure 3). As a result, any variations in the functionality of the orthologous *PYL*/*PYR*/*RCAR*, *PP2C*, and *SnRK2* genes of *S. italica* and *S. viridis* are more likely to manifest at the transcriptional, post-transcriptional, or post-translational levels.

*PYL* receptors of *Setaria* spp. were clustered into the three subfamilies, each containing sequences from both *A. thaliana* and *O. sativa* species (Figure 4). Among the 14 *A. thaliana* PYL receptors, representatives from subfamilies I (PYL1 and PYL3), II (PYL6), and III (PYL8, PYL9, PYL10, and PYL11) triggered the inhibition of PP2C activity in the presence of ABA [36]. Additionally, AtPYL13 inhibited PP2Cs in the ABA-independent pathway, and was able to interact with other PYLs, reducing their activity in the ABA-dependent pathway [15]. The absence of correlation between the tree topology and the function of PYL receptors indicates a complex evolutionary pattern of ABA dependency function. This pattern potentially involves neofunctionalization and functional convergence in the Angiosperm ancestral species.

Comprehending the expression patterns of *PYL* genes is a challenging task. Given that PYL proteins interact with PP2C to activate the SnRK2 signaling cascade, one would anticipate that the genes encoding *PYL* are upregulated under water stress. However, it has been demonstrated, at least in *A. thaliana*, that ABA decreases the expression of six *PYLs* receptors (*AtPYR1*, *AtPYL1*, *AtPYL4*, *AtPYL5*, *AtPYL6*, and *AtPYL8*), while the expression of the remaining receptors exhibits minimal changes [27]. This regulation profile helps prevent a prolonged ABA response, which could potentially harm plant performance. In monocots, several studies have indicated a reduction in the gene expression of *PYL* under ABA-related stresses, with some exceptions. *PYL* genes were reported to be downregulated in the leaves of *S. viridis* during water stress [4] and *Sorghum bicolor* under moisture-deficit stress, except for *SbPYL1* and *SbPYL7*, which were upregulated [37]. Inversely, ABA led to the upregulation of mostly *PYL* loci in the shoots and roots of rice and wheat [28,29]. Our findings indicate that *S. italica* displays a *PYL* gene expression profile under osmotic stress that aligns more closely with other C4 Poaceae species, such as *S. viridis* and *S. bicolor*, as well as *A. thaliana*, than with C3 Poaceae species like rice and wheat.

The *PP2C* phylogenetic analysis enabled the differentiation of two groups of *Setaria* spp. genes: one closely related to *ABIs*, *HABs*, and *AHGs*, and the other more closely associated with *HAIs* from *A. thaliana* (Figure 4). ABI, HAB, and AHG PP2Cs are the canonical ABA-responsive PPC2s, with redundant functions in ABA signaling in the interaction with PYL and SnRK2 proteins [38]. On the other hand, HAI PP2Cs participate in the drought stress response by two complementary mechanisms. The primary function of HAI PP2Cs, particularly HAI1, lies in modulating the signaling pathway related to low water potential, which controls the accumulation of proline and osmoregulatory solutes [39,40]. Additionally, due to their restricted interaction with PYL, HAI PP2Cs can maintain their activity and inhibit SnRK2s in the presence of ABA [41]. Understanding the evolutionary relationships between *Setaria* spp. and *A. thaliana PP2C* genes can contribute in selecting candidate genes for additional functional studies on abiotic stress responses in *Setaria* spp. The upregulation of *S. italica PP2Cs* under water stress is in accordance with the findings on closely related species *S. viridis* [4] and sorghum [37], and also in the more distant eudicot species, among them, tomato [42], soybean [6] and grapevine [43]. The most highly expressed *S. italica PP2C* genes (Figure 5) are grouped closely with *A. thaliana HAI* genes in the phylogenetic analysis (Figure 4). Interestingly, the canonical *A. thaliana* PP2Cs, ABI, HAG, and HAB, are clustered with *Setaria* spp. PP2Cs that were less upregulated (Figure 4 and Figure 5). This clustering reinforced the function of *Setaria* spp. PP2C1, PP2C8, PP2C9, PP2C10, and PP2C11 on the ABA-dependent water deficit response.

*SnRK2s* are divided into three subclasses. *Arabidopsis thaliana* Subclass III *SnRK2s* (*AtSnRK2.2*, *AtSnRK2.3*, and *AtSnRK2.6*) are ABA-dependent kinases, which inhibit seed germination and seedling growth by the modulation of stress-responsive genes [44]. Subclass II *SnRK2s* (*AtSnRK2.7* and *AtSnRK2.8*) are activated in response to osmotic stress, albeit to a very minimal extent in response to ABA [45]. Finally, subclass I *SnRK2s* (*AtSnRK2.1*, *AtSnRK2.4*, *AtSnRK2.5*, *AtSnRK2.9*, and *AtSnRK2.10*) contain ABA-unresponsive kinases that are activated under osmotic stress conditions, except for *AtSnRK2.9*, which is not modulated during water deficit response [44]. Using the topology of our *SnRK2* phylogenetic tree, we can suggest categorizing *S. italica* gene clades into the three *SnRK2* subclasses, providing an initial insight into their role in water deficit responses. In terms of gene expression modulation, *SnRK2s* displayed significant variability, which did not align directly with the identified *SnRK2* subclasses in the phylogenetic analysis (Figure 4). However, these variations were in line with the expression patterns previously reported for *S. viridis* [4]. Multiple studies vary in their findings regarding expression modulation within each *SnRK2* subclass. For example, subclass III genes were found to be downregulated in sorghum [37], rice [20], and grape [43]; however, subclass III exhibited increased expression in tomato and wheat under dehydration conditions [42,46]. These findings indicate variability in the gene expression modulation across various plant species, consistent with our own results. Alternatively, these differences in gene modulation patterns among species may stem from experimental designs and biological sampling specificities.

The expression of ABA core components during stress response is tightly regulated and is more intricate than previously understood [4,47]. A negative-feedback mechanism that attenuates the initial ABA response, leading to desensitization to the elevated levels of ABA, includes the downregulation of *PYL* and the upregulation of *PP2C* [18,48], and can explain, in part, our gene expression modulation profiles (Figure 5). The existence of multiple PYR/PYL/RCAR, PP2C, and SnRK2 proteins might indicate that different PP2Cs bind to different PYL receptors to fine-tune a response to the broad range of ABA concentrations and kinases, contributing to a precise and plastic response [49]. As potential target genes for future investigations, it would be interesting to obtain *Setaria* app. lines that either overexpress or knock down drought-responsive *PP2Cs*, *PYLs*, and *SnRK2s*, and evaluate the impact of these genetic modifications on the phenotype of plants under osmotic stress. *Setaria* spp. *PP2C2*, *PP2C3*, *PP2C4*, *PP2C6*, *PP2C7*, and *PP2C12* present promising targets, as they were upregulated during drought and share close phylogenetic relationships with *A. thaliana HAI* phosphatases, which are known to be overexpressed under drought conditions and regulate the accumulation of proline and osmoregulatory solutes at low water potential [41]. Moreover, conducting transactivation assays could provide valuable insights into the interaction among ABA, PYL receptors, and PP2C phosphatases in *Setaria* spp. This approach would facilitate the identification of specific PP2Cs that modulate subclass III kinases (such as SiSnRK2.1, SiSnRK2.2, and SiSnRK2.3), which exhibit close phylogenetic relationships with AtSnRK2.2, AtSnRK2.3, and AtSnRK2.6 kinases and are primarily ABA-responsive proteins [44]. These represent only a few approaches that can help to unravel the intricate network underlying ABA signaling. In summary, our findings shed light on the genetic diversity, evolutionary relationships, and potential roles of the genes implicated in drought responses in *S. italica* and *S. viridis* species. Understanding the complexity of the ABA signaling network is paramount for deciphering the elaborate mechanisms through which drought-tolerant plants detect and respond to external stressors, ultimately contributing both plant biology and strategies for crop improvement.

## 4. Materials and Methods

### 4.1. Mining and Gene Structure Analyses

*PYR/PYL/RCAR*, *PP2C*, and *SnRK2* coding sequences were identified using the BLASTn tool from the Phytozome 13 database [50], *S. italica* v2.2 and *S. viridis* v4.1 genomes, and previously characterized *S. viridis* sequences [4] as queries. Gene and coding sequences, the chromosomal location, presence and location of PFAM domains, the number of amino acids, and transcription orientation were extracted from Phytozome for subsequent analyses. The genomic sequences and CDSs were used to draw schematic diagrams of the exon–intron structures using the Gene Structure Display Server 2.0 platform (GSDS, [51]). Orthology maps were drawn using the PhenoGram v1 tool [52] and the coordinates of the transcription start site (TSS) of each locus. The sequence identity between the nucleotide and amino acid sequences was evaluated using the p-distance method, calculated by the BioEdit v7.2.6.1 tool [53]. Putative promoter sequences were obtained from Phytozome and utilized to annotate ABRE binding sites using PlantCARE v1 [54] and TE insertion profiles using the Censor v2 tool [55], with a cut-off score ≥ 250. In order to compare the putative regulatory regions of each pair of orthologous genes, we established that the putative regulatory sequences correspond to the longest 5′ UTR region of each orthologous pair plus a 2 kb fragment upstream of the TSS of the same gene.

### 4.2. Phylogenetic Reconstructions

Coding sequences were aligned using the Clustal W v1.4 program [56], as implemented by the BioEdit v7.2.6.1 tool [53]. Phylogenetic relationships were reconstructed using the maximum likelihood method and the PhyML v3 program [57], as implemented in the ATGC Montpellier Bioinformatics Platform [57], according to the best distance method evaluated with the ‘Find the Best Model’ tool of the MEGA X v10.2 program [58]. The phylogenetic trees were reconstructed using the General Time Reversible model and five discrete gamma categories. The robustness of the topology was tested using the aLRT SH-like method [57]. Sequences from *A. thaliana* and *O. sativa* [4] were also included in the phylogenetic reconstructions. The tree was visualized and edited in Fig Tree v.1.4.3 [59].

### 4.3. Meta-Analysis of Gene Expression Profile

To understand gene expression patterns of the genes involved in the ABA signaling pathway during water deficit, a title literature search was performed in Google Scholar [31] and PubMed [32] using the keywords “water deficit” OR “drought”, “tolerance” OR “stress” and “setaria” OR “foxtail”. Previously published studies with available *S. italica* and *S. viridis* RNA sequencing data were compiled in a database of expression values (LogFC). Loci were considered as differentially expressed if they met the criteria of FDR-adjusted *p* ≤ 0.05 and the screening cutoff of LogFC ≤ −1.0 or ≥ 1.0. The resulting database was filtered to show genes belonging to *PP2C*, *SnRK2*, and *PYL/PYR/RCAR* gene families. Heatmaps were generated with the ComplexHeatmap package [60] of R Studio v4.3.1 [61], utilizing the Euclidean method for clusterization and the complete method for distance calculation.

## Figures and Tables

**Table 1:** Identification of *S. italica* and *S. viridis PYL/PYR/RCAR*, *PP2C*, and *SnRK2* genes.

Loci	Scaffold	Transcription Sites		Protein Length	PFAM Domain *	Phytozome Gene Identifier
		Start	Stop			
*SiPYL1*	9	36003871	36005478	207	42–192	Seita.9G311900
*SiPYL2*	1	2860240	2862675	201	38–183	Seita.1G030500
*SiPYL3*	4	36338581	36339202	206	47–198	Seita.4G239500
*SiPYL4*	9	48993015	48994206	220	67–213	Seita.9G437300
*SiPYL5*	3	16201029	16202684	204	53–197	Seita.3G207900
*SiPYL6*	5	40592574	40593198	207	48–195	Seita.5G369100
*SiPYL7*	3	4851938	4854302	205	46–192	Seita.3G076200
*SiPYL8*	1	1185260	1188981	211	53–198	Seita.1G013900
*SvPYL1*	9	34761413	34763538	207	48–192	Sevir.9G318000
*SvPYL2*	1	2710051	2713429	201	43–180	Sevir.1G031000
*SvPYL3*	4	35821353	35821973	206	52–198	Sevir.4G251800
*SvPYL4*	9	46321820	46323300	220	70–213	Sevir.9G441100
*SvPYL5*	3	15949957	15951675	204	56–197	Sevir.3G213000
*SvPYL6*	5	39467969	39468592	207	51–194	Sevir.5G374800
*SvPYL7*	3	5043188	5045597	141	47–131	Sevir.3G077900
*SvPYL8*	1	1156602	1160325	211	55–198	Sevir.1G013800
*SiPP2C1*	3	8217300	8220607	451	133–434	Seita.3G121900
*SiPP2C2*	6	375937	378923	444	107–433	Seita.6G005300
*SiPP2C3*	7	4005506	4010019	453	116–442	Seita.7G021400
*SiPP2C4*	2	26789985	26791627	374	76–362	Seita.2G177500
*SiPP2C5*	5	41297870	41300008	401	87–390	Seita.5G379400
*SiPP2C6*	3	17714334	17716534	422	85–411	Seita.3G218800
*SiPP2C7*	9	50584247	50586340	397	71–330	Seita.9G460200
*SiPP2C8*	1	1218094	1222395	358	105–345	Seita.1G014100
*SiPP2C9*	3	12049066	12052126	381	58–364	Seita.3G164700
*SiPP2C10*	5	32027849	32033321	479	176–462	Seita.5G257700
*SiPP2C11*	5	35033197	35034758	226	44–226	Seita.5G294000
*SiPP2C12*	3	9730764	9733216	399	89–376	Seita.3G139000
*SvPP2C1*	3	8481733	8485238	451	176–434	Sevir.3G124100
*SvPP2C2.1*	6	387891	391296	444	113–396	Sevir.6G004900
*SvPP2C2.2*	6	387891	391296	328	132–281	Sevir.6G005000
*SvPP2C3*	7	785451	790598	451	119–410	Sevir.7G004500
*SvPP2C4*	2	25703196	25705262	376	78–364	Sevir.2G184100
*SvPP2C5*	5	40142449	40144716	401	87–390	Sevir.5G384400
*SvPP2C6*	3	16922545	16924778	422	85–411	Sevir.3G223900
*SvPP2C7*	9	47906442	47908435	397	71–330	Sevir.9G463500
*SvPP2C8*	1	1190419	1194718	358	105–345	Sevir.1G014100
*SvPP2C9*	3	11944691	11947945	381	58–364	Sevir.3G168300
*SvPP2C10*	5	25838987	25843846	479	176–462	Sevir.5G211600
*SvPP2C11*	5	33933094	33934782	226	44–226	Sevir.5G296900
*SvPP2C12*	3	9748111	9750887	398	89–376	Sevir.3G141300
*SiSnRK2.1*	9	4716245	4721220	366	28–284	Seita.9G079800
*SiSnRK2.2*	9	11473483	11476494	362	23–279	Seita.9G169200
*SiSnRK2.3*	3	47385988	47389812	374	37–292	Seita.3G369900
*SiSnRK2.4*	9	43841286	43845704	344	4–260	Seita.9G379000
*SiSnRK2.5*	2	45956775	45961725	339	4–260	Seita.2G394500
*SiSnRK2.6*	1	27251817	27256180	454	94–350	Seita.1G190000
*SiSnRK2.7*	7	20312277	20317276	358	4–260	Seita.7G100500
*SiSnRK2.8*	3	157269	159276	380	4–260	Seita.3G003200
*SiSnRK2.9*	9	36623245	36626468	333	5–261	Seita.9G318200
*SiSnRK2.10*	3	19092667	19097929	360	4–260	Seita.3G230400
*SiSnRK2.11*	5	42440115	42444731	362	4–260	Seita.5G395400
*SvSnRK2.1*	9	4714220	4719224	366	28–284	Sevir.9G078200
*SvSnRK2.2*	9	11263105	11266229	362	23–279	Sevir.9G167300
*SvSnRK2.3*	3	46484835	46487553	375	37–293	Sevir.3G387400
*SvSnRK2.4*	9	41982274	41986678	344	4–260	Sevir.9G384100
*SvSnRK2.5*	2	44725651	44730688	339	4–260	Sevir.2G405700
*SvSnRK2.6*	1	26704997	26709453	454	94–350	Sevir.1G194000
*SvSnRK2.7*	7	19178334	19183417	358	4–260	Sevir.7G108300
*SvSnRK2.8*	3	274209	277259	379	4–260	Sevir.3G004100
*SvSnRK2.9*	9	35394567	35397834	333	5–261	Sevir.9G323900
*SvSnRK2.10*	3	18332249	18337532	360	4–260	Sevir.3G235900
*SvSnRK2.11*	5	41261960	41266866	362	4–260	Sevir.5G400900

* PF10604 (Polyketide_cyc2) for PYL/PYR/RCAR, PF00481 (PP2C) for PP2C, and PF00069 (Pkinase) for SnRK2 proteins.

**Table 2:** Sequence identity between *S. italica* and *S. viridis* orthologues.

Loci	Nucleotide			Amino Acids		
	Identity	Substitutions	Indels *	Identity	Substitutions	Indels *
*SiPP2C1*/*SvPP2C1*	99.7%	3	-	99.7%	1	-
*SiPP2C2*/*SvPP2C2.-1*	99.2%	9	-	99.5%	2	-
*SiPP2C2*/*SvPP2C2.2*	67.9%	80	348	67.3%	31	116
*SiPP2C3*/*SvPP2C3*	99.4%	2	6	99.5%	-	2
*SiPP2C4*/*SvPP2C4*	99.2%	3	6	99.2%	1	2
*SiPP2C5*/*SvPP2C5*	99.9%	1	-	100%	-	-
*SiPP2C6*/*SvPP2C6*	99.7%	3	-	100%	-	-
*SiPP2C7*/*SvPP2C7*	100%	-	-	100%	-	-
*SiPP2C8*/*SvPP2C8*	99.8%	2	-	100%	-	-
*SiPP2C9*/*SvPP2C9*	99.4%	6	-	99.2%	3	-
*SiPP2C10*/*SvPP2C10*	99.9%	1	-	100%	-	-
*SiPP2C11*/*SvPP2C11*	99.7%	2	-	99.1%	2	-
*SiPP2C12*/*SvPP2C12*	99.4%	4	3	99.7%	-	1
*SiPYL1*/*SvPYL1*	100%	-	-	100%	-	-
*SiPYL2*/*SvPYL2*	98.6%	2	6	99.0%	-	2
*SiPYL3*/*SvPYL3*	99.8%	1	0	100%	-	-
*SiPYL4*/*SvPYL4*	99.6%	2	-	99.5%	1	-
*SiPYL5*/*SvPYL5*	99.1%	5	-	100%	-	-
*SiPYL6*/*SvPYL6*	99.8%	1	-	100%	-	-
*SiPYL7*/*SvPYL7*	100%	-	-	100%	-	-
*SiPYL8*/*SvPYL8*	99.6%	2	-	100%	-	-
*SiSnRK2.1*/*SvSnRK2.1*	99.9%	1	-	100%	-	-
*SiSnRK2.2*/*SvSnRK2.2*	99.6%	4	-	100%	-	-
*SiSnRK2.3*/*SvSnRK2.3*	99.3%	4	3	99.4%	1	1
*SiSnRK2.4*/*SvSnRK2.4*	99.8%	2	-	100%	-	-
*SiSnRK2.5*/*SvSnRK2.5*	99.7%	3	-	100%	-	-
*SiSnRK2.6*/*SvSnRK2.6*	99.7%	4	-	99.7%	1	-
*SiSnRK2.7*/*SvSnRK2.7*	100%	-	-	100%	-	-
*SiSnRK2.8*/*SvSnRK2.8*	99.6%	1	3	99.7%	-	1
*SiSnRK2.9*/*SvSnRK2.9*	99.9%	1	-	99.7%	1	-
*SiSnRK2.10*/*SvSnRK2.10*	100%	-	-	100%	-	-
*SiSnRK2.11*/*SvSnRK2.11*	100%	-	-	100%	-	-

* Number of coding nucleotides and amino acids involved in indels.

## Data Availability

Data are contained within the article and Supplementary Materials.

## Supplementary Materials

The following supporting information can be downloaded at: https://www.mdpi.com/article/10.3390/plants13111451/s1, Table S1: Genomic locations and length of regulatory sequences, Table S2: Metadata of papers included in meta-analysis, Table S3: LogFC values of meta-analysis samples. Figure S1. PRISMA flow diagram summarizing the steps and results of paper selection on drought stress in *Setaria* spp. The funnel represents the last step of filtering. References [62,63,64,65,66,67,68] are cited in Supplementary Materials.
