# Supplementary material for: Drought Responses in Poaceae: Exploring the Core Components of the ABA Signaling Pathway in Setaria italica and Setaria viridis
Source: Plants (Basel). 2024 May 23;13(11):1451. doi: 10.3390/plants13111451 (PMC11174756; doi:10.3390/plants13111451)
Supplement: Supplementary file 1 [file plants-13-01451-s001.zip › Supplementary Figure S1 prisma chart.pdf]

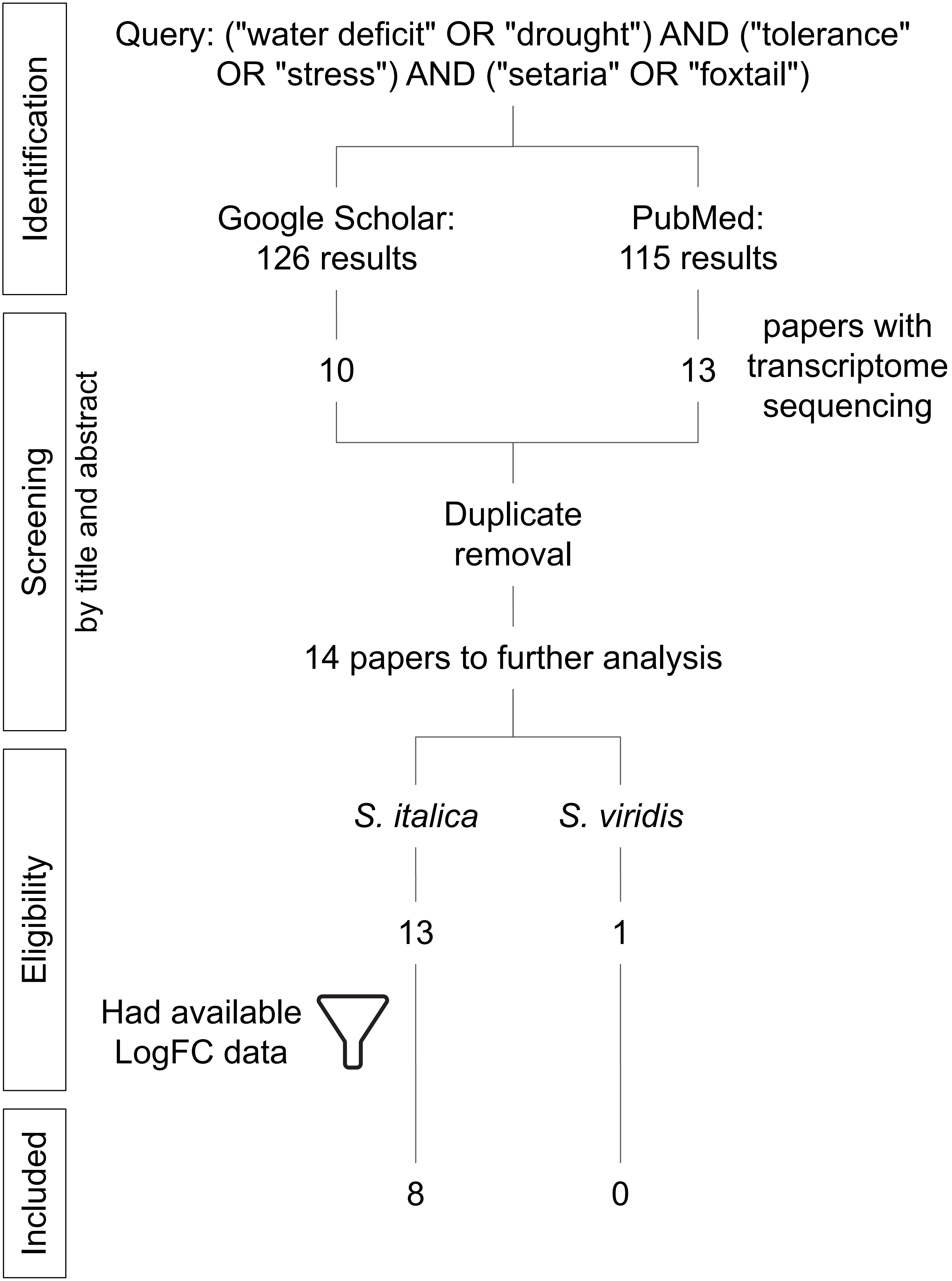
Identification
Screening
by title and abstract
Eligibility
Included
Query: ("water deficit" OR "drought") AND ("tolerance" OR "stress") AND ("setaria" OR "foxtail")
Google Scholar: 126 results
PubMed: 115 results
10
13
papers with transcriptome sequencing
Duplicate removal
14 papers to further analysis
S. italica
S. viridis
13
1
Had available LogFC data
8
0
